# Efficacy of two artemisinin combination therapies for uncomplicated falciparum malaria in children under 5 years, Malakal, Upper Nile, Sudan

**DOI:** 10.1186/1475-2875-4-14

**Published:** 2005-02-24

**Authors:** Ingrid van den Broek, Ribka Amsalu, Manica Balasegaram, Pamela Hepple, Engudaye Alemu, El Badri Hussein, Muhammed Al-Faith, Jacqui Montgomery, Francesco Checchi

**Affiliations:** 1 Manson's Unit, MSF -UK, 67–74 Saffron Hill, London EC1N, UK; 2 MSF-Holland, Khartoum, Sudan; 3 Epicentre, 8 rue Saint-Sabin, 75011 Paris, France; 4 Malawi-Liverpool-Wellcome Trust Clinical Research Programme, Queen Elizabeth Central Hospital, PO Box 30096, Chichiri, Blantyre 3, Malawi

## Abstract

**Background:**

The treatment for *Plasmodium falciparum *malaria in Sudan has been in process of change since 2003. Preceding the change, this study aimed to determine which artemisinin-based combination therapies is more effective to treat uncomplicated malaria in Malakal, Upper Nile, Sudan.

**Methods:**

Clinical trial to assess the efficacy of 2 antimalarial therapies to treat *P. falciparum *infections in children aged 6–59 months, in a period of 42 days after treatment.

**Results:**

A total of 269 children were followed up to 42 days. Artesunate plus Sulfadoxine/Pyrimethamine (AS+SP) and Artesunate plus Amodiaquine (AS+AQ) were both found to be efficacious in curing malaria infections by rapid elimination of parasites and clearance of fever, in preventing recrudescence and suppressing gametocytaemia. The combination of AS+SP appeared slightly more efficacious than AS+AQ, with 4.4% (4/116) versus 15% (17/113) of patients returning with malaria during the 6-week period after treatment (RR = 0.9, 95% CI 0.81–0.96). PCR analysis identified only one recrudescence which, together with one other early treatment failure, gave efficacy rates of 99.0% for AS+AQ (96/97) and 99.1% for AS+SP (112/113). However, PCR results were incomplete and assuming part of the indeterminate samples were recrudescent infections leads to an estimated efficacy ranging 97–98% for AS+SP and 88–95% for AS+AQ.

**Conclusion:**

These results lead to the recommendation of ACT, and specifically AS+SP, for the treatment of uncomplicated falciparum malaria in this area of Sudan. When implemented, ACT efficacy should be monitored in sentinel sites representing different areas of the country.

## Introduction

The health situation in Sudan continues to be affected by long-lasting conflict and related humanitarian emergencies such as food crises and epidemics. Malaria is one of the major causes of morbidity and mortality. In Sudan, an estimated 7.5 million patients suffer from malaria each year and 35,000 die from this disease, which accounts for up to 20% of hospital deaths [1]. The problem appears to have worsened in recent years due to increasing levels of *Plasmodium falciparum *resistance against the two most commonly used antimalarials: chloroquine (CQ) and sulfadoxine/pyrimethamine (SP). CQ resistance in the northern and central part of the Sudan is near 50% [2]. In the South, CQ resistance below 15% was found in some isolated locations [3,4], but was over 80% elsewhere [5,6]. Resistance to SP was shown by presence of SP-resistant genotypes [7] and *in vivo *studies documenting of 0–11% resistance in Khartoum and the eastern part of the country [2,8] and varying levels of 0, 16% up to 70% in different locations in the southern part of the country [4-6]. Amodiaquine (AQ) provided an alternative for CQ in Africa in recent years; this drug is not officially registered in the Sudan, and due to its limited use, was expected to have preserved good efficacy.

The nationally recommended treatment protocol for *P. falciparum *malaria in Sudan at the time of this study was still CQ as first-line and SP as second-line. A change in protocol was however in progress, and the Sudanese Health authorities had indicated that artemisinin-based combination therapy (ACT) was the preferred new first-line treatment [9]. The World Health Organization (WHO), MSF and various other NGOs supported this viewpoint [10,11].

In line with recommendations for several other African countries, two ACTs were proposed for Sudan: Artesunate (AS) +SP or AS+AQ. The third option, used in other African countries, Artemether-Lumefantrine (Coartem) was not seen as a viable option at the time, because of its cost. To provide information on the efficacy of ACT treatment in Sudan, the *in vivo *therapeutic efficacy of the first two combinations were studied in Malakal town, Upper Nile State.

## Patients and Methods

Malakal is located in southern Sudan (on the edge of the Khartoum-governed area), which is an area is of medium to high malaria endemicity (main transmission season: June-November). This open-label study was done in Malakal paediatric hospital supported by Médecins Sans Frontières, from September 2003 to January 2004. The methodology used was in accordance with standard WHO procedures for antimalarial drug efficacy assessment in high transmission settings [12,13]. Children aged between 6 months and 5 years with fever (axillary temperature of 37.5°C or more) and *P. falciparum *infection with a density of 2,000 to 200,000 parasites/μl but no signs of severe malaria (WHO criteria, [13]) or any other serious health condition were included in the study (provided written parental consent was given). One of the two therapies was allocated (by sequential alternation), and given under supervision: either (1) AS+SP, 3 days AS 4 mg/kg body weight on day 0, 1 and 2 plus SP, 25/1.25 mg/kg, single dose on day 0 or (2) AS+AQ, 3 days AS 4 mg/kg on day 0, 1 and 2 plus AQ 10 mg/kg on day 0, 1, 2. Doses were calculated for weight categories in quarter tablets. In case of vomiting within 30 minutes, the treatment was repeated. Repeated vomiting led to exclusion from study and referral to the hospital for quinine treatment. Medications were normally given in the mornings and appointments for next day roughly matched 24 hr intervals. Artesunate was obtained from Dafra (Belgium) and SP and AQ from IDA (the Netherlands).

The sample size of at least 116 inclusions per arm was calculated to detect a 12% difference between the two arms, assuming a 97% efficacy at day 28 for the AS+SP arm (based on 100% efficacy of SP found in a recent study performed at a nearby location, [4]) and 85% for the AS+AQ arm (internationally recommended level for change), with 80% power and 5% risk type I error, anticipating a loss to follow-up of 10%. Patients returned daily during the first 3 days of illness and weekly thereafter for 6 weeks (42 days) after treatment. Side effects of drugs and self-medication were recorded based on patient reports and actively asked for during visits. Cases which remained negative during follow-up were considered Adequate Clinical and Parasitological Responses (ACPR). Failures were classified as (1) Early Treatment Failures (ETF) in case of significant parasitaemia at day 2 or 3 or parasites and fever at day 3, (2) Late Clinical Failures (LCF) for cases with parasites and fever during follow-up after day 3 and (3) Late Parasitological Failures (LPF) for parasite infections without fever at day 42, all in accordance with WHO guidelines [13]. Rescue treatment for failures was quinine 10 mg/kg/eight-hourly for 7 days. PCR analysis was used to distinguish new infections from recrudescences.

Slides were examined by two microscopists independently and 20% of slides were cross-checked in an external laboratory (at KEMRI, Nairobi). The density of parasites was determined by simultaneous count of white blood cells and parasites, assuming a standard density of 8,000 WBCs per μl [13]. Haemoglobin levels were evaluated at day 0, 14, 28 and 42, to observe return to healthy Hb-levels after effective antimalarial treatment. PCR analysis of the *msp1 *and *msp2 *loci was used to distinguish re-infections from recrudescences [14,15]. In case of two or more identical alleles in pre- and post-treatment genotypes, the case was classified as recrudescent, or as a new infection if one or none of the alleles matched. Cases were excluded from the final analysis in case of loss to follow-up, self-medication with antimalarials and concomitant disease and when PCR results showed re-infections or were undetermined due to missing samples, undetermined genotypes or non-amplifiable DNA.

The research protocol was reviewed and approved by the Sudan Research Directorate, the Malaria, Leishmaniasis and Schistosomiasis Directorate, as well as by the Ethical Review Board of MSF (comprising of MSF-external experts only). During recruitment of the children in study, informed written consent was obtained from their parents/guardians.

## Results

**Figure 1:**
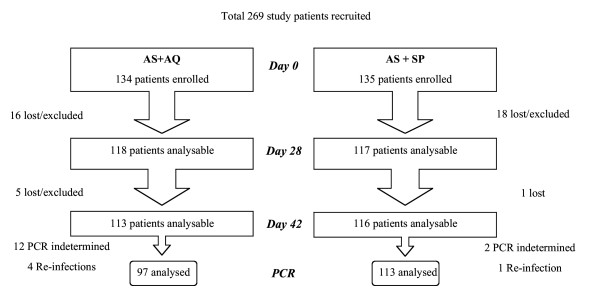
Patient flow during recruitment and follow-up

A total of 269 children were recruited during the 5-month study period. Of these, 134 were treated with AS+AQ and 135 with AS+SP. Baseline characteristics were similar in both treatment groups (Table 1). Of the children included, 40 were excluded from the final analysis due to associated serious other febrile illness (n = 15), self-medication with antimalarials (n = 7), loss to follow-up (n = 13) or erroneous inclusion/classification (n = 5, i.e. three cases of disagreement over microscopy quality control, two cases retreated with parasites but no fever prior to day 42 in AS+AQ group). There were three children with Hb level of 5.0 g/dl included (limit of in/exclusion), based on their condition assessed by the medical doctor. Altogether, 113 (84%) completed the study after treatment with AS+AQ and 116 (86%) with AS+SP (Figure 1).

**Table 1:** Baseline characteristics of patients at study-enrolment, per treatment arm, Malakal, Upper Nile, Sudan 2003–4.

	**AS + AQ **(n = 134)	**AS + SP **(n = 135)
Sex ratio (F/M)	68/66	64/71
Mean age (months) (SD, ranges)	33 (13, 7–59)	33 (15, 6–59)
Mean weight (kg) (SD, ranges)	11.1 (2.4, 5.7–17.0)	11.3 (2.6, 5.4–18.0)
Mean axillary temperature (°C) (SD, ranges)	38.7 (0.9, 37.5–40.9)	38.9 (0.9, 37.5–40.9)
Mean haemoglobin value (g/dl) (SD, ranges)	7.8 (1.7, 5.0–12.2)	7.9 (1.8, 5.0–12.7)
Parasitaemia geometric mean (/μl) (ranges)	20 952 (2 100–199 500)	24835 (2 000–198 000)

Both artemisinin-combitherapies were highly effective to treat *P. falciparum *infections and prevent parasite re-emergence. Only 1 case of ETF was found, in the AS+SP group (Table 2). At day 28 and day 42, the proportion of patients still parasite-free was higher in the group treated with AS+SP than in the group AS +AQ (χ^2 ^test, p = 0.014 for results day 28; p = 0.0049 for day 42).

**Table 2:** Treatment endpoints on day 28, 42 after treatment and PCR adjusted day 42, Malakal, Upper Nile, Sudan, 2003–2004

	**AS + AQ**			**AS + SP**		
	N	%	95% CI	N	%	95% CI
*Day 28 Results*	*117*			*116*		
ACPR	105	**89.7**	82.8–94.6	114	**98.3**	93.9 – 99.8
ETF	0	**0**		1	**0.9**	0.0–4.7
LCF	10	**8.5**	4.2–15.2	1	**0.9**	0.0–4.7
LPF	2	**1.7**	0.2–6.0	0	**0**	

*Day 42 Results*	*113*			*116*		
ACPR	96	**85.0**	77.0–91.0	112	**96.6**	91.4–99.1
ETF	0	**0**		1	**0.9**	0.0–4.7
LCF	15	**13.3**	7.6–20.9	2	**1.7**	0.2–6.1
LPF	2	**1.8**	0.2–6.2	1	**0.9**	0.0–4.7

*PCR corrected day 42*^#^	*97*			*113*		
*No PCR result*	*(12)*			*(2)*		
*Re-infections*	*(4)*			*(1)*		
ACPR	96	**99.0**	94.4–100	112	**99.1**	95.2–100
ETF	0	**0**	0.0–3.7	1	**0.9**	0.0–4.8
LCF	1	**1.0**	0.0–5.6	0	**0**	0.0–3.2
LPF	0	**0**	0.0–3.7	0	**0**	0.0–3.2

^#^Cases with missing or undetermined PCR results and re-infections were excluded from the analysis. ACPR = Adequate Clinical Parasitological Response, ETF = Early Treatment Failure, LCF = Late Clinical Failure, LPF = Late Parasitological Failure.

PCR analysis was unable to generate results for a large part of cases (only 6/20 available, Figure 1). In the AS+AQ treatment group, of 17 failures, only 1 was confirmed to be recrudescent, whereas of the other cases, 4 were re-infections and 12 samples were not analyzable. Of the 3 'failures' in the AS + SP group, one was an early treatment failure, PCR identified 1 re-infection and 2 were non-analysable. Hence, with the PCR correction and exclusions of non-analysable and re-infection cases, AS+AQ resulted in 1.0% confirmed failures (1/97) and AS+SP in 0.9% true failures (1/113) (Table 2). Extrapolation of the cases with known PCR results to the ones that remained undetermined (assuming 2/3 of parasitemia due to reinfection), would give a 95.3% efficacy of AS+AQ (101/106) and 98.2% of AS+SP (112/114). Alternatively, a 'worst case' scenario, assuming that all non-analysable PCRs were actually recrudescences, would lead to 88.1% efficacy in the AS+AQ group (96/109) and 97.4% for AS+SP (111/114).

**Figure 2:**
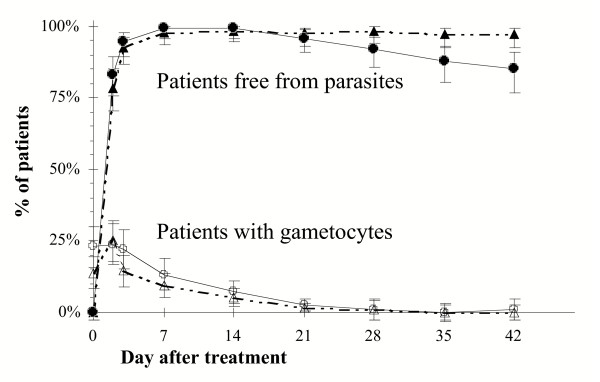
Parasitaemia and gametocytaemia during 6-week follow-up. Symbols: AS+SP ▲ parasites △ gametocytes, AS+AQ ● parasites ○ gametocytes. Error bars indicate 95%CI.

Parasites were rapidly cleared in both treatment groups. At day 2 only 17% and 22% of patients still had a (low) parasitaemia in the AS+AQ and AS+SP groups, respectively. Gametocytes were found in 20% (52/262) of children on inclusion. During follow-up, 78% of these carriers at enrolment showed gametocytes again on one or more follow-up days (32/41), whereas 22% of the children without gametocytes at inclusion were later found to be gametocytemic (42/192). There was no difference between the age, temperature, parasitemia, haemoglobin on admission of the two groups, neither in rates of ACPR at study-endpoint. Gametocyte carriage was similar in both treatment groups and all gametocytes disappeared gradually from the blood during the first weeks after follow-up, and cleared by day 21 in 95% of cases (Figure 2).

The body temperature of all patients went down quickly; fever clearance (T<37.5°C) at day 1 and day 2 was 86% and 97% in the AS + AQ group and 83% and 93% in the AS+SP, and at day 3 all but one (on AS+SP) were free from fever. Average haemoglobin levels for the AS+AQ and AS+SP treatment groups increased from under 8.0 g/dl to near 10 g/dl 6 weeks after treatment. The proportion of children classified as moderately anaemic (haemoglobin from 5 to 8 g/dl) reduced from 51% to 11% during follow-up period, similar in both arms. No serious adverse events were observed or reported on routine clinical examination during follow-up and none of the failures developed complicated malaria.

## Discussion

This is one of the first studies into the clinical outcomes of ACT combination therapy in Sudan and provides useful information for decision-makers working to ensure effective antimalarial protocols in this part of the country.

Both of the artemisinin-based combination therapies tested here were found to be highly efficacious in the treatment of uncomplicated *P. falciparum *malaria in this area of the Sudan. AS+SP appears to be the better treatment option on the basis of non-PCR corrected responses, showing a lower percentage of patients returning with parasitaemia. The PCR analysis indicates the true efficacy is comparable between both treatments (near 99%), but is, however, limited by a high proportion of indeterminate cases. More realistically, an efficacy between 97–98% can be expected for AS+SP and 88–95% for AS+AQ, acceptable levels after the long follow-up of 42 days after treatment. A rapid parasite clearance and fever reduction was found following treatment with both ACTs. The rise in haemoglobin values and the reduction of the proportion of (moderately) anaemic children after treatment confirms that the malaria parasites were effectively removed from the blood and red blood cell levels rose after treatment.

The ACTs tested also had an effect on gametocytes. In general, the gametocidal action of AS appears to work through preventing the development of new gametocytes rather than clearance of existing ones [16]. In our study, the 20% of gametocyte-carrying infections at enrolment cleared gradually by day 21. Newly detected gametocytes developed in 22% of cases after treatment, which is lower than after monotherapy, at least for SP. A previous study in Upper Nile [4] showed that 68% of patients (of all ages) treated with SP and 28% of those treated with AQ developed gametocytaemia during the 14 days after treatment, while gametocyte prevalence at admission was only 2%. Gametocidal effect is very important since the sexual stages of parasites are essential for person-to-person transmission of malaria via mosquito vectors.

The main limitations of the study were that the number of patient exclusions were higher than anticipated (15%) due to concomitant febrile illnesses and loss to follow-up, as well as the lack of results for PCR-analysis, caused by samples missing or an inability to provide sufficient DNA on amplification due to low densities in post-treatment samples. Increasing the number of inclusions compensated losses to follow-up. Repeated attempts at PCR-analysis for problematic samples were only partly successful and, therefore, lead to extrapolation about the findings for the missing samples.

At time of writing, a change of national protocols towards ACT in northern Sudan is in preparation – coordinated among health authorities, NGOs and other relevant actors. The first line treatment recommended country-wide is AS+SP, based on reported high efficacy of SP. Northern Sudan is the only country in Africa, which has chosen the option AS+SP [17]. On the basis of the results of this study, this is justifiable. One other study in Sudan on AS+SP and AS+AQ efficacy was recently completed in the Nuba Mountains and also shows high efficacy of 91.2% and 92.7%, respectively, for these ACTs at 28 days after treatment [18]. ACTs with SP or AQ as companion drugs have shown to be very effective in other areas of Africa, provided that the companion drug still maintained a good level of efficacy [19-22]. In the Upper Nile area both SP and AQ were still an effective treatment for *P. falciparum *and SP has shown high efficacy in various areas of northern Sudan [2,4,23]. It remains to be seen whether AS+SP will be equally efficacious in other, e.g. more northern areas of the Sudan. In Southern Sudan AS+AQ has recently been put forward as the therapy of choice [17]. The use of two different therapies would seem a sensible option in a vast country with areas of different patterns of resistance.

An advantage of AS+SP over AS+AQ is that it is more convenient to administer, as SP is given as a single dose and can be administered under observation in a health facility, whereas AQ requires 3 days to complete a course. SP tablets are also easier to take (AQ has a bitter taste). Blister packs of AS+SP, which combine the two drugs and clearly indicate daily tablets to be taken, are currently available for different age categories in Sudan. AS+SP is cheaper than AS+AQ.

Implementation of the new national protocol with AS+SP will hopefully take place as quickly as possible to prevent rising morbidity and mortality. Vulnerable displaced populations in epidemic-prone areas and areas of high perennial malaria transmission should be prioritized. Introduction of ACT will have to go hand in hand with laboratory confirmed diagnosis (microscopy or rapid diagnostic tests) to prevent unnecessary use of valuable drugs (thus minimizing drug pressure) and ensure that non-malarial cases are appropriately treated. The change in guidelines should also filter through all health service providers, including the private sector and drug vendors to decrease the potential risk of SP monotherapy and incomplete dosages. The change will initially require more funds to be made available for malaria treatment. The international community – including many NGOs, the WHO, donors, and the Global Fund – has shown willingness to support countries to change antimalarial protocols [24,25].

At present, MSF has already started to pilot AS+SP treatment in its project areas in Northern Sudan, i.e. Darfur, Upper Nile and Gedaref, on behalf of the Ministry of Health. The implementation of treatment as well as the future efficacy of AS+SP should be monitored carefully in a number of dispersed sentinel sites, as there is a possibility that SP resistance may further rise before the combination has been made available countrywide.

## Authors' contributions

IB was responsible for overall supervision, data analysis and writing of the paper, RA for field-implementation, communication as well as data analysis and writing, MB for preparation and set-up of study, support and advocacy. PH was responsible for microscopy laboratory work and quality. EA participated as field supervisor of clinical work and data gathering. EBH and MAF were responsible for day-to-day study procedures and supervision of clinical teams. JM did the PCR analysis and FC was involved in design of study, data analysis and writing of manuscript. All authors read and approved the final manuscript.
